# Overexpression of Sucrose Phosphate Synthase Enhanced Sucrose Content and Biomass Production in Transgenic Sugarcane

**DOI:** 10.3390/plants9020200

**Published:** 2020-02-06

**Authors:** Risky Mulana Anur, Nurul Mufithah, Widhi Dyah Sawitri, Hitoshi Sakakibara, Bambang Sugiharto

**Affiliations:** 1 Center for Development of Advanced Science and Technology (CDAST), University of Jember, Jember 68121, Indonesia; risky.max@gmail.com (R.M.A.); mufithah1018@gmail.com (N.M.); widhi.d.s@ugm.ac.id (W.D.S.); 2 Present address: Department of Agronomy, Faculty of Agriculture, University of Gadjahmada, Yogyakarta 55281, Indonesia; 3 RIKEN Center for Sustainable Resource Sciences, Yokohama 230-0045, Japan; sakaki@agr.nagoya-u.ac.jp; 4 Graduate School of Bioagricultural Sciences, Nagoya University, Nagoya 464-8601, Japan; 5 Department of Biology, Faculty of Mathematic and Natural Science, University of Jember, Jember 68121, Indonesia

**Keywords:** biomass, sucrose, soluble acid invertase, sucrose phosphate synthase, transgenic sugarcane

## Abstract

Sucrose phosphate synthase (SPS) is a key enzyme in sucrose synthesis, which controls sucrose content in plants. This study was designed to examine the efficacy of the overexpression of *SoSPS1* gene on sucrose accumulation and carbon partitioning in transgenic sugarcane. The overexpression of *SoSPS1* gene increased SPS activity and sucrose content in transgenic sugarcane leaves. More importantly, the overexpression enhanced soluble acid invertase (SAI) activity concomitant with the increase of glucose and fructose levels in the leaves, whereas sucrose synthase activity exhibited almost no change. In the stalk, a similar correlation was observed, but a higher correlation was noted between SPS activity and sugar content. These results suggest that SPS overexpression has both direct and indirect effects on sugar concentration and SAI activity in sugarcane. In addition, SPS overexpression resulted in a significant increase in plant height and stalk number in some transgenic lines compared to those in non-transgenic control. Taken together, these results strongly suggest that enhancing SPS activity is a useful strategy for improving sugarcane yield.

## 1. Introduction

Sugarcane (*Saccharum officinarum*), a C4 plant, is a major crop for sucrose production in tropical and sub-tropical areas. Sucrose is synthesized via photosynthesis in the leaf, after which it is transported to, and accumulates in, the stalk. In general, sucrose metabolism in plants involves several enzymes, such as sucrose phosphate synthase (SPS; EC 2.4.2.14), sucrose synthase (SuSy; EC 2.4.1.13), and invertase (EC 3.2.1.26). SPS is a key enzyme for sucrose synthesis from uridine diphosphate-glucose (UDPG) and fructose-6 phosphate (F6P). SuSy catalyzes reversible reactions: either synthesis or cleavage of sucrose with UDPG and fructose; it is mostly present in non-growing sink tissue and plays a role in the sucrose degradation pathway [1]. There are several isoforms of invertase, the major ones being the vacuolar and cell wall invertases that cleave sucrose to glucose and fructose under weak acidic conditions (pH 4.5 to 5.0), which are called soluble acid invertase (SAI). Plants also have invertases with optimal pH at neutral and slightly alkaline ranges, but they are rather minor and less characterized [2]. In sugarcane, the net sucrose accumulation in the stalk depends on the balance between sucrose synthesis by SPS and the breakdown activities by SAI [3,4].

Genes-encoding SPS have been cloned from various plants, including maize [5], *Arabidopsis* [6], and sugarcane [7,8]. The presence of SPS isoform has also been reported in plants such as sugarcane [7] and *Arabidopsis* [6] with different expression patterns. There are two SPS isoforms in sugarcane: *SoSPS1* that is expressed in photosynthetic tissue and *SoSPS2* that is constitutively expressed in all tissue [7]. To date, many studies were conducted in order to understand the role of SPS in sucrose accumulation. It was reported that the overexpression of *SPS* increased the sucrose:starch ratio and the photosynthetic rate in the leaves of transgenic tomato [5,9] and *Arabidopsis thaliana* [10]. Another study showed that *SPS* overexpression resulted in increased sucrose unloading in tomato fruit [11]. It was also shown that the overexpression of *SPS* affected carbon partitioning and carbohydrate metabolism. Constitutive overexpression of *SPS* increased sucrose synthesis in older leaves and accelerated whole plant growth in transgenic tobacco [6,12]. Effects on plant growth and biomass by *SPS* overexpression have also been examined in transgenic *Arabidopsis* and poplar [13], *Brachypodium distachyon* [8], and tobacco [6]. However, the effect of SPS activity elevation on sucrose content and growth in sugarcane, which accumulates a large amount of sucrose in the sink stalk, has not yet been successfully characterized.

The involvement of invertase in the control of sucrose content and plant growth was also reported. Exogenous sucrose supplies increase invertase activity in sugarcane [14,15]. The overexpression of invertases accelerate sucrose hydrolysis and enhance plant growth in cotton, *Arabidopsis*, and loquat [16,17]. On the other hand, the downregulation of SAI by foliar chemical treatment or the inhibition of SAI activity increases sucrose content in sugarcane [18,19]. Efforts were made to reduce invertase activity using antisense techniques, but there was no significant increase in the yield of sucrose in sugarcane [20].

The knowledge of the role of SuSy in sucrose accumulation and usage is rather limited. It was thought that sucrose provides substrate for cellulose synthesis via the action of SuSy, which catalyzes sucrose cleavage to generate UDPG. The downregulation of a cucumber sucrose synthase 4 (CsSUS4) suppressed the growth and development of flowers and fruit in conjunction with low hexose, starch, and cellulose content [21]. However, the involvement of SuSy in sucrose accumulation in sugarcane is not fully characterized. Sucrose metabolism is organized under a complex regulation of SPS, SAI, and SuSy. Therefore, the characterization of these enzyme activities in genetically modified sugarcane, together with sugar accumulation and growth traits, is important for a better understanding of sugar metabolism in the sugar crop. In this study, a sugarcane *SPS* gene (*SoSPS1*) is overexpressed under the control of CaMV 35S promoter in sugarcane. We characterized the effect on SPS, SAI, and SuSy activities, sugar content, and plant growth. Our results show that increasing SPS activity is an effective strategy for enhancing the sucrose content and growth of the sugar crop.

## 2. Results

### 2.1. Expression of SoSPS1 Gene in Transgenic Sugarcane

The selected lateral buds from the first generation of transgenic sugarcane were grown in a greenhouse for six months. To confirm the insertion of the transgene of pBI121-*SoSPS1* construct, genome DNA was isolated from the leaves of one-month-old transgenic and non-transgenic (NT) sugarcane and subjected to PCR analysis. The PCR analysis showed the amplification of 0.55 kb *npt*II DNA in three independent transgenic lines, but not in the NT line (Figure S2). We also confirmed a single hybridization band of the *npt*II transgene in a Southern blot analysis (Figure S3). These results show that the transgene was properly inserted into the sugarcane genome.

**Figure 1:**
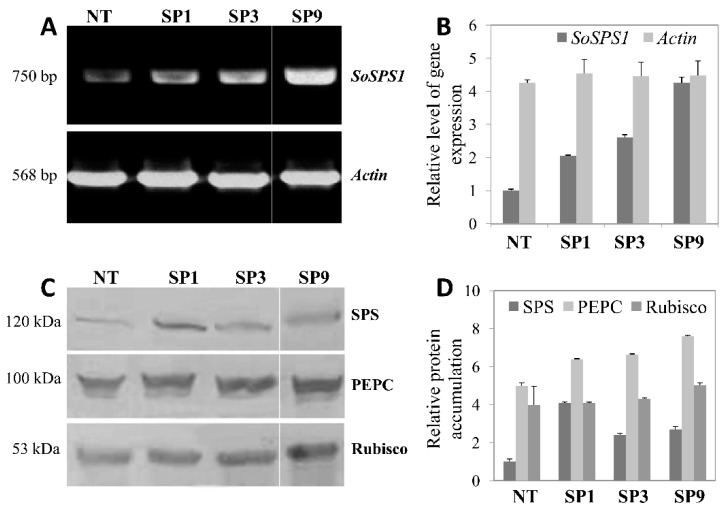
Expression of sucrose phosphate synthase (SPS), phosphoenolpyruvate carboxylase (PEPC), and ribulose-1,5-bisphosphate carboxylase/oxygenase (Rubisco) in the leaf of non-transgenic (NT) and transgenic sugarcane lines (SP1, SP3, SP9). (**A**) Transcript levels of *SoSPS1* and *Actin* (reference control) in the sugarcane lines as determined by RT-PCR. Cycle numbers in PCR were 25 and 20 min for *SoSPS1* and *Actin*, respectively. (**C**) Protein levels of SPS, PEPC, and Rubisco-large subunit (LSU) detected by immuno-blotting. (**B**,**D**) Intensities of the amplified cDNA and protein bands analyzed by ImageJ free software (https://imagej.nih.gov/). The results are expressed as relative values of the control NT (=1.0). Fully expanded two-month-cultivated sugarcane leaves were harvested at daytime and divided into two parts for RNA and protein extraction. One microgram of total RNA was reverse-transcribed to first strand cDNA and used for PCR. Then, 30, 10, and 5 µg of total soluble proteins were subjected for immunoblot analysis for SPS, PEPC, and Rubisco-LSU proteins, respectively.

The transcript levels of *SoSPS1* gene were determined by semi-quantitative RT-PCR. The results show that the accumulation level increased in all transgenic lines compared to the NT. The expression levels of *SoSPS1* transcript in SP9 was highest among the transgenic lines. On the other hand, the accumulation of *Actin* transcript used as a control was almost at the same level in all of the lines examined (Figure 1A,B). These results suggest that the increased *SoSPS1* transcripts were caused by the overexpression of *SoSPS1* transgene.

The accumulation of SPS protein in the transgenic sugarcane leaves was analyzed by immunoblot. Proteins were detected at around 120 kDa, corresponding to sugarcane SPS. As we observed in the RT-PCR analysis, the detected protein level in transgenic lines was higher than that in NT (Figure 1C,D). The accumulation pattern of SPS transcript and that of SPS polypeptide in NT and transgenic lines were basically correlated, except for SP1 (Figure 1). This might be due to post-transcriptional effects, such as translation efficiency or protein stability. In comparison, phosphoenolpyruvate carboxylase (PEPC) protein levels showed slight increases, but no increase was exhibited by the ribulose-1,5-bisphosphate carboxylase/oxygenase (Rubisco)-large subunit (LSU) protein in the transgenic lines (Figure 1C,D). A similar result was also reported in the C3-type PEPC of transgenic alfalfa overexpressing a maize *SPS* gene [22].

### 2.2. Sucrose Metabolizing Enzymes Activities

**Figure 2:**
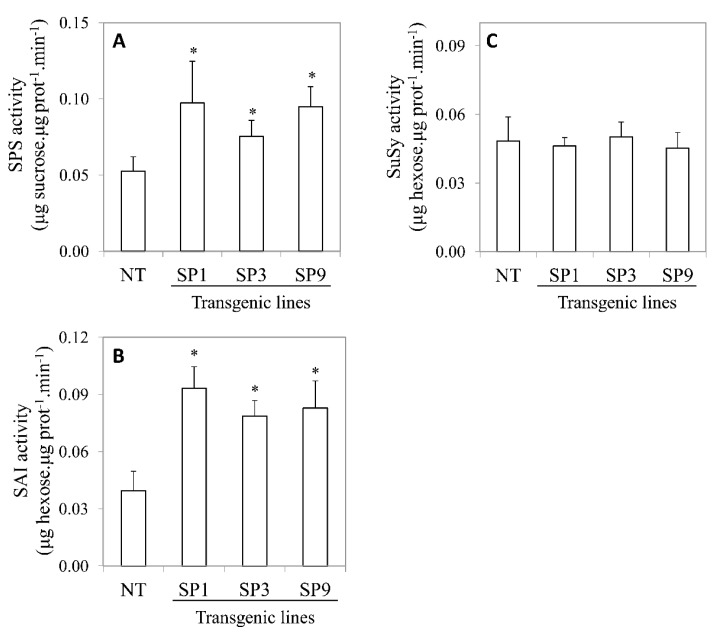
Activities of SPS (**A**), soluble acid invertase (SAI) (**B**), and sucrose synthase (SuSy) (**C**) in leaves of NT and transgenic sugarcane lines (SP1, SP3, SP9). Total soluble protein was extracted from fully expanded sugarcane leaves as described in the legend of Figure 1. The activities of enzymes were measured as described in Section 4. Values are means ± SD for three independent plants. Asterisks denote statistically significant differences (*t*-test: *p* < 0.05).

The measurement of SPS activity showed an enhancement in transgenic sugarcane compared to NT sugarcane (Figure 2A). The higher SPS activity appears to be observably correlated with SPS protein levels detected by immunoblot analysis (Figure 1C,D). The SPS activities in the SP1 and SP9 lines were increased approximately two-fold compared to NT sugarcane. Thus, the overexpression of *SoSPS1* gene resulted in increasing protein levels, as well as SPS activities in transgenic sugarcane. Interestingly, this increase was accompanied by significant increases in SAI activities (Figure 2B). On the other hand, SuSy activities were not affected (Figure 2C). These results suggest that enhancing SPS activity increases SAI activity in sugarcane.

### 2.3. Increasing Sugar Content in the Leaves and Stalks of Transgenic Sugarcane

To determine the effect of enhanced SPS activity on sugar accumulation, the sucrose, glucose, and fructose contents were measured in the leaves and stalks of the sugarcane lines. Compared to the NT line, the sucrose content of the leaves of transgenic lines increased (Table 1). The accumulation of fructose and glucose also increased in the transgenic lines, probably due to rising SAI activities. The hexose content increased at a higher rate than the sucrose content. The highest hexose content increased by 12-fold, and the sucrose content only increased by 2.4-fold in the leaves of transgenic lines. When the SPS activity was compared to sucrose levels, the correlation coefficient was low (0.05) (Figure S4A). On the other hand, hexose levels in the leaves exhibited a strong positive correlation with SAI activity, with coefficients of 0.52 and 0.77 for glucose and fructose, respectively (Figure S4B). The low correlation coefficient between SPS and sucrose content suggests that sucrose synthesized by SPS could not accumulate in the leaves of transgenic sugarcane and was immediately degraded or exported to other organs.

In the stalks, the sucrose content in transgenic lines also significantly increased by 1.3- to 1.4-fold (Table 1). When the sucrose content was compared with SPS activity, a positive correlation was found, with a coefficient of 0.42 (Figure S4C). This result suggests that the enhancement of SPS activity increases the unloaded sucrose accumulation in the stalks of sugarcane. On the other hand, the unloaded sucrose was partially hydrolyzed for metabolism, since the glucose and fructose content also increased by 1.3- to 1.9-fold in the stalks of transgenic lines, but the increase rates were lower than that in the leaves (Table 1).

### 2.4. The Effect of SPS Overexpression on Sugarcane Growth

To know the effect of *SPS* overexpression on sugarcane growth, the transgenic sugarcane lines grown for six months were harvested, and agronomical traits (plant height, stalk diameter, stalk number, and stalk weight) were investigated. These traits in the transgenic lines showed that overexpression of *SoSPS1* gene significantly increased plant height, and also had a positive effect on stalk growth (Table 2). The overexpression significantly increased stalk numbers in the SP3 and SP9 lines and stalk weight per pot in the SP3 transgenic line. However, the overexpression did not affect the stalk diameter of the transgenic lines (Table 2). The positive correlation coefficient between SPS activity and sugarcane height was 0.71 (Figure S4E). Total stalk weight is an important determinant for sugarcane productivity. Thus, a combination of the higher sucrose content and total stalk weight could estimate that sugar production increased in transgenic sugarcane.

## 3. Discussion

We demonstrate that overexpression of *SoSPS1* gene in sugarcane increased the accumulation of SPS protein and its activity, leading to sucrose accumulation and increased biomass. The leaf SPS activity increased by 1.4- to 1.9-fold, followed by increased sugar content in the leaves and stalks of transgenic lines (Table 1). A positive correlation coefficient was found between leaf SPS activity and sucrose content in stalks; however, such a correlation was not found in the leaves. This suggests that sucrose could not efficiently accumulate in the leaves and should either be cleaved or translocated to sink organs. Given that sucrose supply could induce invertase activity in sugarcane [14,15], a part of the sucrose could be cleaved by the increased SAI activity to produce hexose for energy provision for growth. Recently, the roles of SPS in sucrose metabolism and plant growth were reported. The overexpression of *SPS* resulted in an increased yield of transgenic potatoes [23], altered growth and development in transgenic tobacco [6,24], and improved biomass production in *B. distachyon* [8]. Similarly, SPS overexpression in sugarcane not only increased sucrose content, but also improved growth traits, such as plant height, number of stalks, and stalk weight per pot (Table 2); hence, the total sugar production is expected to increase.

Several studies showed that sucrose accumulation inhibits photosynthesis [25,26,27], and exogenous sucrose supply strongly reduces the net CO_2_ assimilation in sugarcane [14]. The results obtained in this study show that the overexpression of *SoSPS1* results in increased sucrose content concomitant with increased sucrose degrading invertase activity in the leaves. The increase in sucrose degrading activity might play a role in modulating sucrose levels so as not to exceed the level of photosynthesis gene suppression. Therefore, the effect of sucrose levels on gene suppression will be examined in the next experiment on transgenic plants. Similarly, exogenous sucrose was shown to alter acid and neutral invertase activities in sugarcane [14]. These results support a model in which the sucrose-cleaving enzymes play a pivotal role in maintaining the balance between sucrose signaling and metabolism [28,29]. Sugar-related metabolism is linked to plant development, and the abundance of hexose induces cell division and expansion [30,31]. Thus, increased biomass accumulation in transgenic sugarcane may be a result of complex mechanisms.

Sugarcane accumulates a high concentration of sucrose in the stalk, but the mechanism for highly efficient translocation and accumulation remains unclear. In most plants, sucrose synthesized in the leaves is exported to sink organs mediated by a sucrose transporter and/or SWEET proteins. Several studies have shown that the overexpression of a sucrose transporter gene increased sucrose unloading and sink strength [32,33,34,35,36]. SWEET can transport sucrose across the plasma membrane in various plants, such as *Arabidopsis* [37], sorghum [38], and *Lotus japonicus* [39]. *SWEET* expression is essential for sugar efflux for pathogen nutrition [40] and the cooperation between sucrose synthesis by SPS and SWEET is required for nectar secretion [41]. Thus, it is postulated that the manipulation of sucrose transporter genes, as well as *SWEET* expression, in cooperation with increased SPS activity, might further increase sucrose concentration in the stalks of sugarcane. In addition, it was recently reported that N-terminal truncated SPS shows higher activity, avoiding regulation by allosteric effectors [42]. Future research will aim at further increasing sucrose accumulation in plants using the N-terminal deleted *SPS*.

## 4. Materials and Methods

### 4.1. Plant Transformation and Growth Condition

*Agrobacterium*-mediated transformation of sugarcane was initiated by constructing *SoSPS1*-cDNA in a binary vector of pBI121 (Takara, Shiga, Japan). The full length of *SoSPS1*-cDNA [7] was inserted into the binary vector driven by a 35S promoter (Figure S1). The cDNA construct was prepared by amplification of the cDNA using a forward primer containing an additional *Spe*I site (F4) and a reverse primer with a *Spe*I site (R4) (Table 3). The amplified cDNA was digested with the *Spe*I (*Xba*I compatible) and inserted into the *Xba*I site of GUS-removed pBI121 plasmid. Sugarcane in vitro shoots were used as explant for *Agrobacterium* transformation according to the method previously described [43]. The sugarcane shoot was prepared by micropropagation of meristematic apical tissue isolated from 4 to 5 months of sugarcane growth in the field of Bululawang (BL) cultivars. The green and healthy shoots (100 explants) were excised around 0.2–0.3 cm from the base, collected, injured using needles, and used as the materials for the transformation. The injured shoots were then co-cultivated with *Agrobacterium tumefaciens* harboring the pBI121-*SoSPS1* in the presence of 100 ppm of acetosyringone. After three days of co-cultivation in a dark room, the infected sugarcane shoots were incubated in Murashige and Skoog (MS) basal media containing cefotaxime (500 mg L^−1^) for a week with illumination, followed by incubation in MS media containing antibiotic kanamycin (50 mg L^−1^) and cefotaxime (500 mg L^−1^) for three weeks. The surviving shoots were sub-cultured in the same selection media and, after five successive cycles, the surviving putative transformants were acclimated in a growth chamber. The transformation was carried out in a three-time independent experiment and the putative transformants were combined for analysis. The transformation efficiency was around 6%. The negative control of non-transgenic (NT) sugarcane was cultured in MS media without *Agrobacterium* infection and antibiotic selection.

The acclimated sugarcane plantlets were transferred to 15 L pots containing a mixture of soil/sand/organic matter (50:25:25) in the greenhouse for vegetative propagation in the Center for Development of Advanced Science and Technology, University of Jember. The light intensity of the greenhouse was approximately 650 µmol m^−2^ s^−1^ at the plant level. The humidity and temperature were adapted to the ambient conditions ranging from 70% to 80% RH (relative humidity) and 24 (day) to 30 °C (night), respectively. The second generation of vegetatively propagated lateral buds were germinated and grown in 15 L pots with the same mixture, and then randomly placed in the greenhouse for six months. Each sugarcane line was cultivated in three biological replicates. Growth traits such as the number of tillers and internodes, plant height, and biomass were measured at the harvest. For molecular and biochemical analysis, fully expanded sugarcane leaves were harvested at the indicated time and plunged into liquid nitrogen. The results were statistically evaluated by Dunnett’s test and *t*-test at *p* ≤ 0.05.

### 4.2. Genomic and Gene Expression Analysis

Genomic DNA was isolated from 3 g sugarcane leaves, as previously described [43], and stored at −20 °C until analysis. The presence of the inserted gene of interest was analyzed by PCR using the genomic DNA and a pair of primers for the detection of *npt*II gene (Table 3). The PCR reaction was performed in a T100 thermal cycler (Bio-Rad, Irvine, CA, USA) and the PCR product was separated in 1% (w/v) agarose gel, then documented with GelDoc (Major Science, Saratoga, California, USA). To confirm the presence of gene insertion, a Southern blot analysis was performed using genomic DNA. The genomic DNA (20 µg) was digested with restriction enzyme of *Hind*III and separated in 1% agarose gel electrophoresis. The separated DNA was then transferred to a nitrocellulose membrane (Hybond N+, 3-) and hybridized with a DIG-labeled DNA probe of *npt*II gene according to the manufacturer’s instructions (Roche, Mannheim, Germany).

Gene expression analysis was conducted by the detection of *SoSPS1* gene transcript using RT-PCR analysis. Total RNA was extracted from 0.5 g of frozen sugarcane leaves using a kit for RNA isolation (Tiangen, Beijing, China). The RNA content was measured with a NanoVue spectrophotometer (GE Healthcare, Piscataway, NJ, USA). One microgram (µg) of total RNA was converted into cDNA using reverse transcriptase (RT) and oligo-dT primer (Roche, Mannheim, Germany). The first strand cDNA was used for the detection of *SoSPS1* gene transcript by semi-quantitative RT-PCR using a primer pair of F2–R2 (Table 3). The *Actin* expression was determined using a primer pair of F3–R3 (Table 3) and was used as the reference expression gene. The reactions were carried out in the T100 thermal cycler (Bio-Rad, Irvine, California, USA) with 25 and 20 cycles for the detection of *SoSPS1* and *Actin* transcripts, respectively. The amplified DNAs were separated in 1% agarose gel electrophoresis and visualized with GelDoc (Major Science, Saratoga, CA, USA).

### 4.3. Protein Extraction, Enzyme Assay, and Immunoblotting

Frozen sugarcane leaves (1 g) were pulverized in liquid nitrogen, and the frozen powder was continuously ground in three-time volumes (w/v) of extraction buffer containing 50 mM 3-morpholinopropane sulfonic acid (MOPS)-NaOH (pH 7.5), 10 mM MgCl_2_, 1 mM ethylenediaminetetraacetic acid (EDTA), 2.5 mM dithiothreitol (DTT), and 1 mM phenylmethanesulfonyl fluoride (PMSF) in the presence of 10% polyvinylpyrrolidone (PVP). The leaf homogenates were centrifuged at 14,000× *g* at 4 °C for 10 min. The partial supernatant (crude extract) was desalted using gel filtration of Sephadex G-25 (GE Healthcare, Piscataway, NJ, USA) equilibrated with the extraction buffer, and then used for enzyme activity measurements. The remaining crude extract was stored at −80 °C until immunoblotting analysis. Protein concentration was determined using a reagent of Bradford (Bio-Rad, Des Plaines, IL, USA).

SPS activity was assayed by measuring the formation of sucrose-6-phosphate in the desalted extract as previously described [42]. The assay mixture (70 µL) contained 30 mM MOPS-NaOH (pH 7.5), 10 mM MgCl_2_, 15 mM UDP-glucose, 10 mM fructose-6-phosphate (F6P), and 10 mM glucose-6-phosphate (G6P). The reaction was initiated by adding 30 µL of desalted leaf extract, incubated at 30 °C for 10 min. It was terminated by adding 70 µL of 1 M NaOH. The remaining unreacted F6P was destroyed by incubating at 95 °C for 10 min, and after chilling on ice, 0.25 mL resorcinol (1%) and 0.75 mL of 30% HCl were added. The mixture was incubated at 80 °C for 8 min and the developed color was measured using a spectrophotometer at 520 nm. The SPS activity in the leaf was calculated as the quantity of sucrose produced per minute at 30 °C.

SAI activity was measured according to a previously described method [4] with a little modification. The 50 μL desalted leaf extract was added to 50 μL reaction mixture containing 1 M sodium acetate buffer (pH 4.5) and 0.25 M of sucrose, and was incubated at 37 °C for 30 min. The reaction was terminated by adding 30 µL of 2.5 M Tris base, and then incubated at 95 °C for 3 min. SuSy activity was determined by the sucrose cleave direction according to a previously reported method [44] with a little modification. The 30 µL desalted extract was added to a 70 µL reaction mixture containing 20 mM Tris-HCl (pH 7.0), 100 mM sucrose, and 4 mM UDP, and was incubated at 37 °C for 30 min. The reaction was terminated by heating to 95 °C for 5 min. The content of reducing sugar produced during the reactions of SAI and SuSy was determined using a 3:5-dinitrosalicylic acid (DNS) reagent with a spectrophotometer at 540 nm [45]. SAI and SuSy activities in the leaves were calculated as the quantity of reducing sugars produced per minute at 37 °C.

Immunoblot analysis was directed to measure the levels of phosphoenolpyruvate carboxylase (PEPC), ribulose-1,5-bisphosphate carboxylase/oxygenase (Rubisco) large subunit (LSU), and SPS proteins in sugarcane leaves. The analysis was conducted by separating the proteins from the crude extract using SDS-PAGE (12.5% polyacrylamide) and transferring them onto the Immobilon-P transfer membrane (Thermo Scientific, Rockford, IL, USA) using a semi-dry trans-blotter (Bio-Rad, Irvine, CA, USA). The membrane was then separately incubated with polyclonal antibodies against PEPC, Rubisco [46], or recombinant SPS1 proteins [42], and then diluted in tris-buffered saline (TBS) containing 0.5% skim milk overnight. After washing with TBS, the membrane was incubated with a secondary antibody of goat anti-rabbit IgG Alkaline phosphatase conjugate (Bio-Rad, Irvine, CA, USA) at 1:3000 dilution for 60 min. The reacted bands of PEPC, Rubisco, or SPS1 proteins were visualized by incubating the membrane with a mixture of the substrate, 5-bromo-4-chloro 3-indolyl-phosphate (BCIP), and nitroblue tetrazolium (NBT) (Bio-Rad, Irvine, CA, USA).

### 4.4. Sugar Analysis

Frozen leaf material (2 g) was ground in a mortar with liquid nitrogen, followed by continuous grinding in a 5 mL mixture of methanol:chlorofom:water (12:5:3, v/v/v). After centrifugation of the extract at 5000× *g*, the pellet was rinsed again with the mixture, and the supernatant fractions from five successive washes were combined. The combined supernatants were concentrated to dryness with a rotary-evaporator at 40 °C and the residues were dissolved in a fixed amount of distilled water. Undissolved material was removed by centrifugation, and the supernatant was stored at −20 °C until sugar analysis. Sugarcane juice was extracted from the sugarcane stalk, centrifuged, and stored at −20 °C until sugar analysis. The sucrose, glucose, and fructose contents were determined by high-performance liquid chromatography (HPLC) (Hitachi, Tokyo, Japan) with a reflection index detector at 40 °C. After passing through a 0.22 µm Millipore filter, the soluble sugars were separated on a reverse-phase column of Shimadzu NH2 (4.6 mm internal diameter × 250 mm length) with a mixture of acetonitrile and aquadest (85:15, v/v) at a flow rate of 1 mL min^−1^. Sugar content was expressed as mg/g fresh weight (FW).

## Figures and Tables

**Table 1:** Sugar content in leaves and stalks of NT and transgenic lines (SP1, SP3, SP9). Sugars were extracted from the leaves and stalks of 6-month-grown sugarcane and measured using high-performance liquid chromatography (HPLC). Values are means ± SD for three independent plants, and the different lowercase letters denote significant differences (ANOVA, Dunnett’s test, *p* ≤ 0.05). FW represents fresh weight.

Lines	Leaf Tissue			Stalk Tissue		
	Sucrose (mg/g FW)	Fructose (mg/g FW)	Glucose (mg/g FW)	Sucrose (mg/g FW)	Fructose (mg/g FW)	Glucose (mg/g FW)
NT	2.27 ± 0.10 c	0.35 ± 0.17 b	0.18 ± 0.06 b	71.07 ± 3.30 b	2.33 ± 0.31 b	3.23 ± 1.02 c
SP1	3.59 ± 0.04 b	3.34 ± 0.39 a	2.21 ± 0.58 a	80.40 ± 8.32 b	2.87 ± 0.46 ab	4.40 ± 1.15 bc
SP3	5.51 ± 0.24 a	3.38 ± 0.58 a	1.52 ± 0.64 ab	94.23 ± 3.34 a	3.62 ± 0.20 a	6.25 ± 1.06 a
SP9	3.02 ± 0.34 b	2.47 ± 0.08 a	1.30 ± 0.83 ab	98.52 ± 5.55 a	3.31 ± 0.26 ab	4.86 ± 0.30 ab

**Table 2:** Growth performance of NT and transgenic lines (SP1, SP3, SP9) in a greenhouse for 6 months. Stalk weight measured after removing all leaves from part of the plant. Values are mean ± SD for three independent plants and the different lowercase letters denote significant differences (ANOVA, Dunnett’s test, *p* ≤ 0.05).

Lines	Plant Height (cm)	Stalk Diameter (cm)	Stalk Number	Stalk Weight per Pot (g)
NT	99.67 ± 3.67 b	2.24 ± 0.03 b	9.00 ± 1.00 b	3537.90 ± 680 b
SP1	115.33 ± 6.00 a	2.38 ± 0.03 a	10.67 ± 1.53 ab	4193.06 ± 600 ab
SP3	112.78 ± 5.01 a	2.24 ± 0.04 b	12.67 ± 0.58 a	4979.26 ± 226 a
SP9	124.33 ± 3.93 a	2.18 ± 0.05 b	11.67 ± 0.58 a	4586.16 ± 226 ab

**Table 3:** List of primers used.

Primer Names	Sequence (5′–3′)	Product (bp)	Target Genes
F1	TGAATGAACTGCAGGACGAG	550	*npt II*
R1	AGCCAACGTATGTCCTGAT	550	*npt II*
F2	TGAAGGACACACCGGCAGATG	750	*SoSPS1*
R2	CTTTGATGAGGAAGGCGAAGC	750	*SoSPS1*
F3	GCAACTGGGATGACATGGAG	568	*Actin*
R3	ATGGCTGGAAGAGGACCTCAG	568	*Actin*
F4	TGCACTAGTCGCCCTTCCCA	3425	*SoSPS1*
R4	TCCACTAGTAACGGCCGCCA	3425	*SoSPS1*

## Supplementary Materials

The following are available online at https://www.mdpi.com/2223-7747/9/2/200/s1, Figure S1: Schematic diagram of pBI121-*SoSPS1* construct. Full-length *SoSPS1*-cDNA was inserted into the pBI121 plasmid as described in Section 4. CaMV 35S promoter, Cauliflower mosaic virus 35S promoter; NOS terminator, nopaline synthase gene terminator; NOS promoter, nopaline synthase gene promoter; NeoR/KanR, neomycin phosphotransferase gene (kanamycin resistance gene); RB and LB, T-DNA right and left border, respectively; Figure S2: PCR amplification of *npt*II gene (*NPT*) from genomic DNA of NT and transgenic sugarcane lines. The genomic DNA was isolated from leaves of one-month-grown sugarcane. The amplified DNA with F1–R1 primers (Table 1) was separated in agarose gel electrophoresis and photographed; Figure S3: Southern blot analysis of sugarcane leaf genomic DNA. Southern blot analysis was carried out according to the method described in Section 4. SP1, SP3, and SP9 were transgenic lines, and NT was a non-transgenic line; Figure S4: Relationship between SPS and SAI activities and sugar content and growth traits (*n* = 12). (A) Correlation between SPS activity and sugar content in the leaves, (B) correlation between SAI activity and sugar content in the leaves, (C) correlation between SPS activity and sugar content in the stalks, (D) correlation between SAI activity and sugar content in the stalks, (E) correlation between SPS activity and plant height, and stalk number and weight, (F) correlation between SAI activity and plant height, and stalk number and weight.
