# Supplementary material for: Overexpression of Sucrose Phosphate Synthase Enhanced Sucrose Content and Biomass Production in Transgenic Sugarcane
Source: Plants (Basel). 2020 Feb 6;9(2):200. doi: 10.3390/plants9020200 (PMC7076389; doi:10.3390/plants9020200)
Supplement: Supplementary file 1 [file plants-09-00200-s001.pdf]

**Supplement 1**

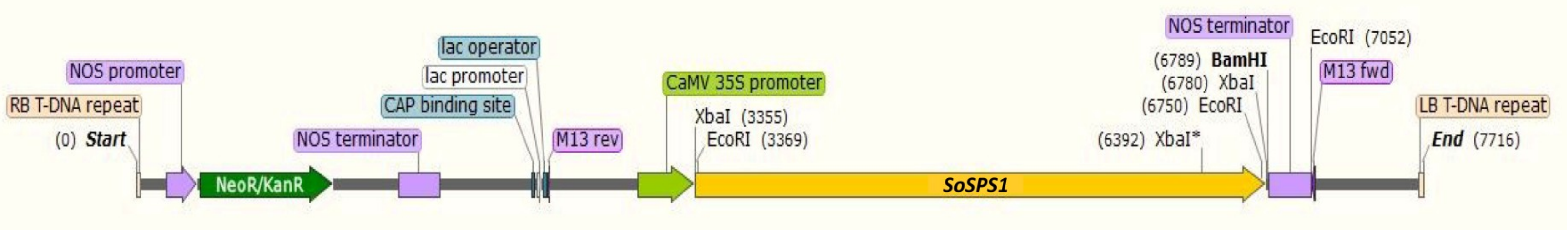


Schematic diagram of pBI121-*SoSPS1* construct. Full-length *SoSPS1*-cDNA was inserted into pBI121 plasmid as described in "materials and methods" section. CaMV 35S promoter, Cauliflower mosaic virus 35S promoter; NOS terminator, nopaline synthase gene terminator; NOS promoter, nopaline synthase gene promoter; NeoR/KanR, neomycin phosphotransferase gene (kanamycin resistance gene); RB and LB, T-DNA right and left border.

**Supplement 2**

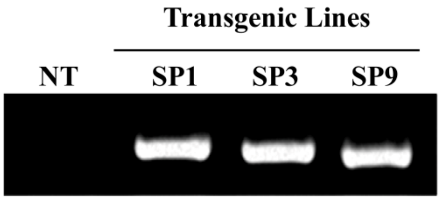


PCR amplification of *npt*II gene (*NPT*) from genomic DNA of NT and transgenic sugarcane lines. The genomic DNA was isolated from leaves of one-month grown sugarcane. The amplified DNA with F1-R1 primers (Table 1) were separated in agarose gel electrophoresis and photographed.

**Supplement 3**

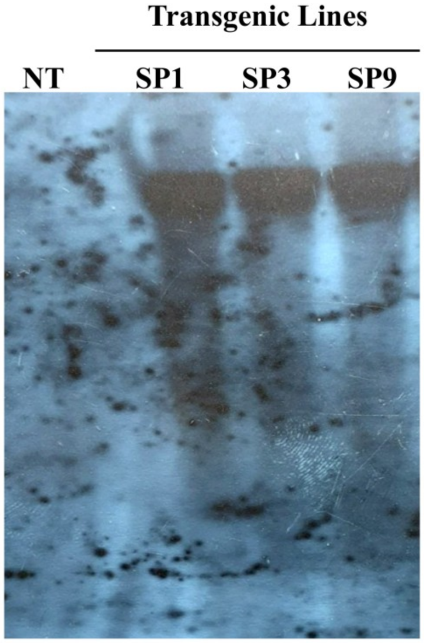


Southern blot analysis of sugarcane leaf genomic DNA. Southern blot analysis was carried out according to the method described in "materials and methods" section. SP1, SP3, SP9 were transgenic lines, and NT was non transgenic line.

**Supplement 4**

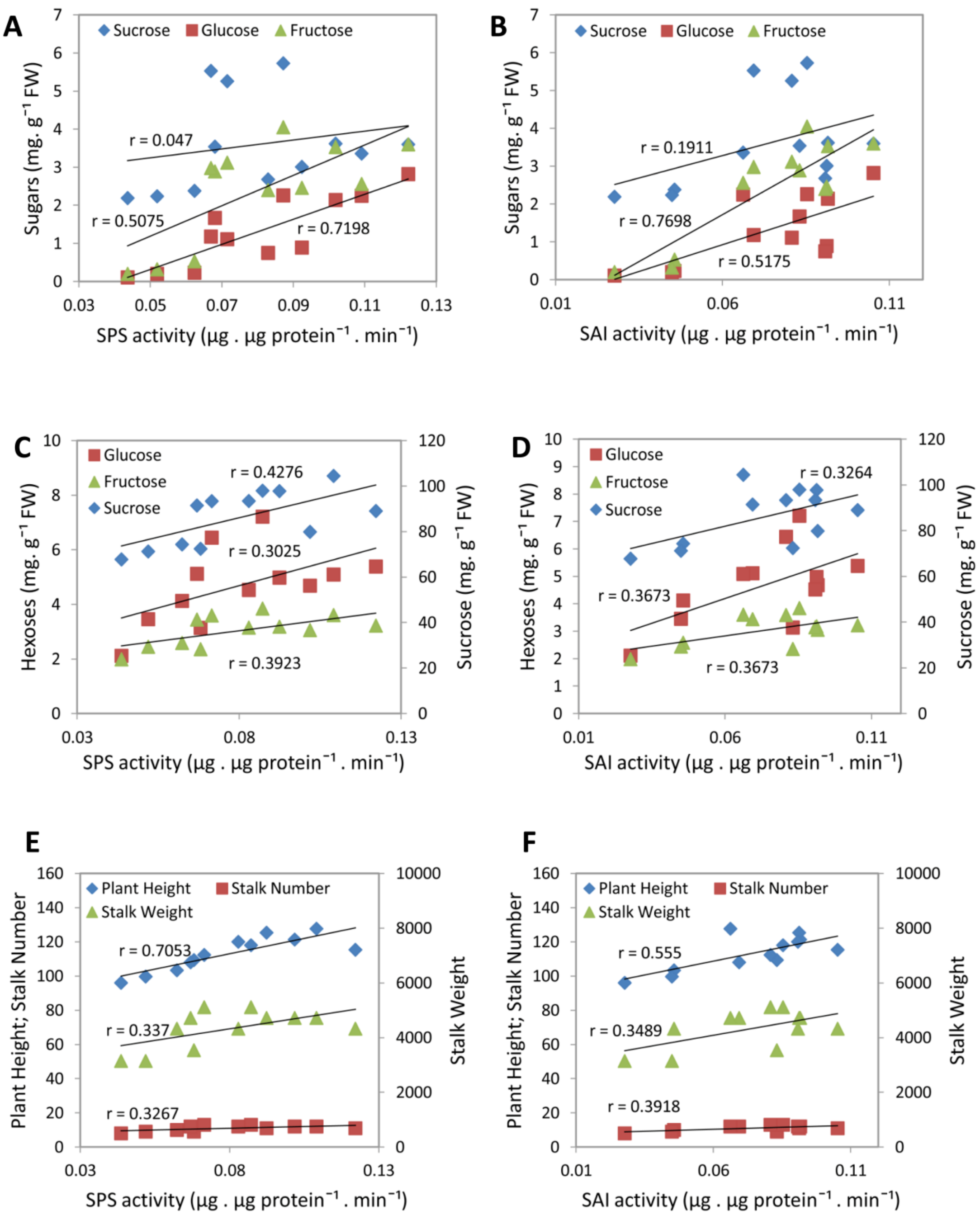


Relationship between SPS and SAI activities in leaf and sugar content and growth traits. (A) Correlation between SPS activity and sugar content in leaf, (B) Correlation between SAI activity and sugar content in leaf, (C) Correlation between SPS activity and sugar content in stalk, (D) Correlation between SAI activity and sugar content in stalk, (E) Correlation between SPS activity and plant height, and stalk number and weight, (F) Correlation between SAI activity and plant height, and stalk number and weight.
